# Network‐Pluralistic Psychiatry in Epilepsy: A theoretical framework and research agenda for personalized, mechanistic, and integrated care

**DOI:** 10.1002/pcn5.70237

**Published:** 2025-11-06

**Authors:** Ryouhei Ishii

**Affiliations:** ^1^ Department of Occupational Therapy Osaka Metropolitan University Graduate School of Rehabilitation Science Habikino Osaka Japan; ^2^ Department of Rehabilitation Osaka Kawasaki Rehabilitation University Kaizuka Osaka Japan; ^3^ Department of Psychiatry Osaka University Graduate School of Medicine Suita Osaka Japan

**Keywords:** collaborative care, Default Mode Network (DMN), epilepsy, polygenic risk, psychiatric comorbidity, Salience Network (SN)

## Abstract

Psychiatric comorbidities in epilepsy are prevalent, disabling, and persistently undertreated due to conceptual and operational fragmentation between neurology and psychiatry. This paper proposes Network‐Pluralistic Psychiatry in Epilepsy (NPPE), a hypothesis‐generating, disease‐contextualized framework that integrates four mechanistic layers: (1) shared genetic vulnerability, (2) epileptogenic network dysfunction as a neuropsychiatric substrate, (3) a bidirectional psychosocial stress loop, and (4) societal and institutional amplifiers. NPPE moves beyond high‐level biopsychosocial heuristics by positing testable causal pathways linking genetic liability to network instability, symptom expression, and environmental feedback in epilepsy. The manuscript situates NPPE within existing integrated‐care paradigms (e.g., collaborative and stepped‐care models), proposing NPPE as a mechanistic engine to personalize measurement‐based treatment‐to‐target decisions while explicitly acknowledging feasibility constraints for advanced tools. A tiered assessment concept (Tiers 1–3) and candidate mechanism‐informed interventions are outlined as research targets rather than clinical recommendations. The paper delineates a research agenda prioritizing longitudinal, multimodal cohorts; incorporation of network biomarkers in clinical trials; evaluation of integrated‐care implementations; and attention to equity and real‐world feasibility. NPPE aims to provide a coherent, testable roadmap to transform descriptive comorbidity accounts into mechanism‐oriented, personalized neuropsychiatric care in epilepsy, contingent on rigorous validation, refinement, or falsification.

## Graphical abstract

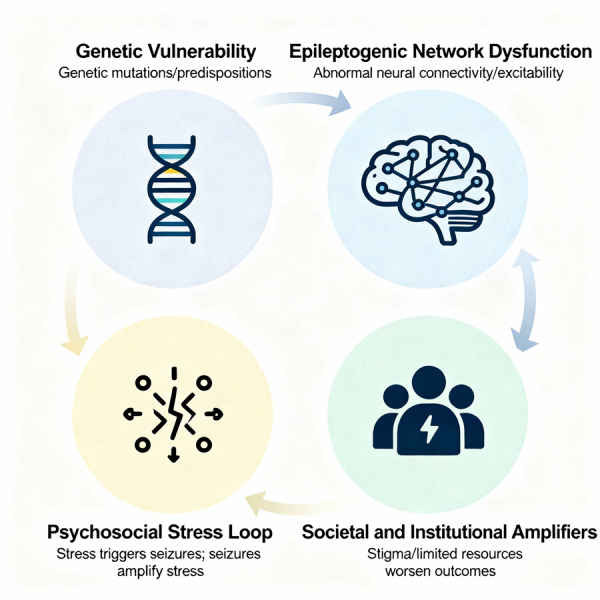

## INTRODUCTION: THE CRITICAL FAILURE IN EPILEPSY CARE

Epilepsy is one of humanity's oldest documented afflictions, with descriptions dating back to ancient Babylon, yet it remains profoundly misunderstood even in the 21st century.^1^ For millennia, it occupied a liminal space between the spiritual and the physical, the divine and the pathological.^2^ In the modern era, this liminality persists—not as superstition, but as the institutional and intellectual chasm that separates neurology from psychiatry. While neurology has claimed the seizure—the electrical storm in the brain—as its undisputed domain, the vast and turbulent landscape of the epileptic mind, with its storms of mood, thought, and identity, has become a neglected borderland. It is a territory divided by two often non‐communicating intellectual states, leaving the person with epilepsy stranded in a no‐man's‐land of fragmented care.^3^

A crucial question arises: Are these psychiatric comorbidities merely the nonspecific consequences of living with a chronic neurological illness, or do they possess features distinctive to epilepsy? The answer is both. Certainly, people with epilepsy share with individuals with other central nervous system (CNS) disorders (e.g., multiple sclerosis, Parkinson's disease) a high burden of reactive depression and anxiety stemming from chronic illness, disability, and social stigma. However, the psychiatric landscape in epilepsy is distinguished by several unique features that demand a disease‐specific framework. These include the following: (1) a well‐documented bidirectional relationship, where psychiatric distress can lower the seizure threshold and seizures can precipitate psychiatric symptoms; (2) the existence of peri‐ictal phenomena, in which psychiatric symptoms are temporally locked to the seizure cycle, such as ictal fear, postictal depression, or the dramatic presentation of postictal psychosis; and (3) the occurrence of specific interictal syndromes like the Schizophrenia‐Like Psychosis of Epilepsy (SLPE), which has a distinct phenomenology compared to idiopathic schizophrenia.^1^, ^2^, ^3^ These epilepsy‐specific phenomena underscore why generic biopsychosocial (BPS) models are insufficient.

The consequences of this fragmentation present significant challenges to patient well‐being and clinical outcomes. A person with epilepsy is not merely a brain prone to seizures; they are a whole person whose life, identity, and mental well‐being are inextricably linked to the neurobiological reality of their condition. The prevalence of depression, anxiety, psychosis, and suicide among people with epilepsy is not a matter of minor comorbidity but constitutes a staggering public health crisis, with depression affecting up to 50% of patients in tertiary care centers and suicide risk several times that of the general population.^1^, ^4^, ^5^ Yet, the dominant paradigms of care often prove inadequate, leading to what can be described as a clinical and conceptual impasse.

This paralysis stems from a dual failure of thought. On one hand, the field is crippled by dogmatism, manifesting as two opposing, yet equally damaging orthodoxies: a neurological reductionism that views all psychic distress as a mere epiphenomenon of aberrant electrical activity, and a psychiatric formalism that attempts to force the complex neuropsychiatric reality of epilepsy into preexisting diagnostic boxes, ignoring the unique etiological context.^1^, ^6^ On the other hand, where these dogmas fail, the field collapses into atheoretical eclecticism—a pragmatic but conceptually limited approach characterized by haphazard polypharmacy and a patchwork of psychosocial interventions applied without a coherent mechanistic rationale.^7^, ^8^

This paper will argue that this state of affairs is no longer tenable. The siloed, dualistic approach to epilepsy care is a relic of 20th‐century thought that has been rendered obsolete by the revolutions in genetics and network neuroscience.^8^, ^9^ The time has come for a new, integrated paradigm. Drawing on the principles of network pluralism, we present Network‐Pluralistic Psychiatry in Epilepsy (NPPE) as a hypothesis‐generating framework, not a validated clinical protocol. Its clinical implications are provisional and require rigorous empirical testing before implementation.^8^ This paradigm moves beyond the simplistic mind–brain divide, conceptualizing the psychiatric dimensions of epilepsy as an emergent property of a dynamic, multilayered system. It posits that psychiatric symptoms arise from a causal cascade that spans (1) a shared genetic foundation, (2) the instability of specific, overlapping brain networks, (3) a bidirectional vortex of psychosocial stress, and (4) the amplifying pressures of a fractured societal and institutional system. By articulating this multi‐level, mechanistic model, NPPE offers a conceptual path to escape the twin traps of dogmatism and eclecticism. It provides a proposed framework whose clinical utility must be rigorously tested, but which holds the potential to pave the way for a truly integrated, personalized, and scientifically grounded approach to care for one of the world's most vulnerable patient populations.

## A CRITICAL APPRAISAL OF THE STATUS QUO—THE ARCHITECTURE OF FAILURE

The inadequacy of the current approach to the psychiatric comorbidities of epilepsy is not a result of isolated clinical errors but of a deeply entrenched, systemic failure in the conceptual architecture of care.^3^, ^4^ This architecture is built upon the flawed foundations of dogmatism and is shored up by the unstable scaffolding of eclecticism.^6^

### Discipline divided: The neurological–psychiatric schism

The intellectual landscape of epilepsy care is dominated by two competing orthodoxies that exist in a state of mutual incomprehension.^10^ This schism perpetuates a dualism that often fails to adequately address the patient's holistic needs, whose lived experience recognizes no such neat division between mind and brain.^1^, ^6^

#### The dogma of neurological reductionism: The brain without a mind

Rooted in the 19th‐century triumphs of neuropathology and the 20th‐century diagnostic power of the electroencephalogram (EEG), neurological practice has, for decades, privileged a model of epilepsy as a fundamentally electrical disorder.^11^ Within this paradigm, the primary goal is the classification and suppression of seizures. The patient's subjective experience of distress—their anxiety, their depression, their cognitive fog—is often relegated to the status of a secondary concern, an epiphenomenon to be explained away within a narrow biological framework.^1^

This reductionist dogma manifests in several ways. First is the pervasive use of the term “organic” to describe psychiatric symptoms. A patient's profound existential despair may be labeled “postictal depression,” or their chronic, debilitating anxiety dismissed as an “interictal phenomenon.” While these labels contain a kernel of neurobiological truth, their application often serves to foreclose deeper inquiry.^10^ The term “organic” can become a clinical stop sign, effectively communicating that the patient's suffering is a direct, unmediated product of brain dysfunction, rendering psychological exploration unnecessary.^6^ A patient describing feelings of worthlessness and hopelessness following a seizure cluster is not just experiencing a “postictal state”; they are grappling with the terror of losing bodily autonomy, the shame of public exposure, and the fear of brain damage, all of which are amplified by a transient neurochemical imbalance.^3^ The reductionist lens sees only the imbalance, not the person experiencing it.

Second, this dogma leads to a clinical culture where seizure frequency becomes the paramount metric of treatment success, often at the expense of quality of life (QOL).^10^, ^12^ A patient may be rendered seizure‐free by an aggressive medication regimen, a clear victory from a purely neurological perspective. However, if that same regimen leaves them with crushing fatigue, cognitive slowing, and emotional blunting, have they truly been made well? The neurological dogma struggles with this question because it lacks the conceptual tools to weigh seizure counts against subjective well‐being.^3^ The result is a clinical encounter focused on the seizure diary, while the patient's silent despair goes unaddressed.^1^

This is not to denigrate the importance of seizure control, which is a vital and often life‐saving goal. The critique is aimed at the intellectual framework that sees seizure control as the only goal, reducing the complex human experience of living with epilepsy to a set of electrical variables to be manipulated.^10^

#### The dogma of psychiatric labeling: The mind without a brain

On the other side of the chasm stands the dogma of psychiatric formalism. When a person with epilepsy is referred to a psychiatrist, they often enter a world that, while attentive to their subjective suffering, is equally blind to its unique neurobiological context.^1^ The psychiatrist's primary tool is the diagnostic manual (DSM or ICD), a system of classification based on symptom clusters, largely agnostic to etiology.^13^

This approach creates its own set of problems. The patient's experience is forced into prefabricated diagnostic boxes that may not fit. For instance, the SLPE is a well‐documented phenomenon, particularly in temporal lobe epilepsy (TLE).^14^ Yet, applying the standard criteria for schizophrenia can be profoundly misleading. The phenomenology of SLPE is often distinct from idiopathic schizophrenia, notably characterized by a preservation of warm affect and fewer negative symptoms, and it follows a different clinical course.^15^ Its neurobiological substrate is intimately linked to the epileptogenic network in the limbic system, a fact that is lost when it is simply labeled “schizophrenia.”^11^ This mislabeling can lead to inappropriate treatment, as antipsychotics may lower the seizure threshold, and the underlying epileptic process goes unaddressed.^10^

Similarly, diagnosing “Major Depressive Disorder” in a person with TLE using standard criteria is an exercise in decontextualization.^1^ The patient's anhedonia is not arising in a vacuum; it may be directly linked to the dysfunction of the brain's reward circuits within the temporal lobe.^3^ Their feelings of hopelessness are a rational response to facing a lifetime of seizures, social stigma, and cognitive challenges.^16^ Treating this as identical to idiopathic depression, without accounting for the unique contributions of the epileptic network, medication side effects, and psychosocial reality, is a profound clinical error.^1^

The core failure of this dogma is its inability to integrate etiology into diagnosis and treatment. It sees the mind as a “black box” that produces symptoms, just as the neurological dogma sees the brain as a “black box” that produces seizures.^6^ The crucial, bidirectional link between the two remains in shadow.^1^

### Integrating NPPE within existing care frameworks

Our critique of fragmentation should not be misconstrued as a dismissal of the significant progress made in integrated care. The need for coordinated services is well‐recognized, leading to the development of evidence‐based frameworks such as the Collaborative Care Model (CCM) and stepped‐care approaches, which have proven effective in other chronic diseases^17^ and are being adapted for epilepsy.^18^

However, a limitation of many current integrated‐care models is that their intervention algorithms, while evidence‐based, can be mechanistically generic. For instance, in a typical stepped‐care protocol, a patient with non‐improving depression might be offered a selective serotonin reuptake inhibitor (SSRI) or cognitive behavior therapy (CBT), but the choice is often guided by general principles rather than a deep understanding of the individual's specific drivers of distress. This is the conceptual and clinical gap that NPPE is designed to address.

The question, therefore, is not whether to replace these proven models, but how to enhance them. NPPE can be operationally embedded as a “mechanistic engine” inside the chassis of these existing structures. A high Patient Health Questionnaire‐9 (PHQ‐9) score would still be the trigger for action, but it would prompt a mechanism‐based inquiry guided by the four layers. This multi‐level understanding transforms the “treatment‐to‐target” decision. The intervention is no longer a generic “next step” in an algorithm but a highly personalized strategy. The NPPE‐informed team might prioritize a switch in antiepileptic drugs (AEDs) over adding an SSRI, or focus on connecting the patient with vocational rehabilitation services rather than standard talk therapy. This approach would also change the roles within the CCM team: The psychiatric consultant's advice would be grounded in network pharmacology, and the care manager's tasks would expand to track not just symptom scores but also progress on psychosocial and systemic barriers.

In this vision, NPPE does not compete with CCM. Instead, it serves as the mechanistic engine inside the high‐performance chassis of CCM. By fusing the structural strengths of established integrated‐care models with the conceptual precision of NPPE, we can move from efficient care to genuinely integrated and personalized neuropsychiatric practice.

## A NEW PARADIGM—NPPE

To escape the intellectual cul‐de‐sac of dogmatism and eclecticism, we must move beyond a linear, single‐cause framework and embrace the logic of complex systems. The NPPE offers such a framework. It reframes the psychiatric comorbidities of epilepsy not as a separate “illness” or a simple “symptom,” but as an emergent property of a multi‐level, dynamically interacting system that is under stress.^8^, ^19^

This paradigm is pluralistic because it recognizes that causality operates simultaneously at multiple levels, from genes to social structures. It is a network paradigm because it focuses on the connections and interactions between these levels, rather than viewing them as isolated silos.^8^, ^9^ The NPPE model proposes a four‐layer causal cascade, providing a comprehensive roadmap to understand why a particular individual with epilepsy develops a specific psychiatric profile.

### Layer 1: The shared genetic foundation—The initial blueprint of vulnerability

The traditional view posits epilepsy and mental illness as distinct entities that can co‐occur. The NPPE paradigm begins with a more radical premise: In many cases, epilepsy and its psychiatric comorbidities are not two separate diseases, but are divergent clinical manifestations of a single, underlying genetic vulnerability.^20^ The foundation of psychiatric suffering in epilepsy is often laid down in the genome itself.

This understanding is built upon decades of research in molecular genetics. Early on, studies of rare, single‐gene disorders provided a proof of principle. For example, mutations in genes like SCN1A, which code for a sodium channel subunit, can cause severe epileptic encephalopathies like Dravet syndrome, which is almost universally accompanied by profound cognitive and behavioral impairments.^21^ More recently, large‐scale, genome‐wide association studies (GWAS) have revealed a more complex and widespread picture for common epilepsies and psychiatric disorders. These studies have shown that conditions like idiopathic generalized epilepsy, focal epilepsy, schizophrenia, and bipolar disorder do not have a single “gene for” them. Instead, their risk is conferred by the cumulative impact of hundreds or thousands of common genetic variants (polymorphisms), each with a tiny individual effect.^22^

Crucially, the genetic architectures of these disorders overlap significantly. A landmark study from the Brainstorm Consortium (2018), for example, analyzed the genomes of over a million individuals and found significant genetic correlations between epilepsy and major depression, Attention‐Deficit/Hyperactivity Disorder, and schizophrenia.^23^ This is not a statistical curiosity; it is a window into a shared biology. Many of the implicated genes converge on a handful of fundamental neurodevelopmental processes: ion channel function (governing neuronal excitability), synaptic plasticity (the basis of learning and network formation), and immune regulation.^24^

A gene like CACNA1C, for instance, which codes for a calcium channel subunit, has been identified as a risk gene for bipolar disorder, schizophrenia, and epilepsy.^25^ This makes biological sense: Calcium channels are fundamental to regulating neuronal firing and neurotransmitter release. A variant that makes these channels slightly dysfunctional could plausibly increase the risk of both uncontrolled, synchronized firing (a seizure) and the more subtle dysregulation of neural circuits that underpins mood disorders. This concept of pleiotropy, where one gene influences multiple, seemingly unrelated traits, is a cornerstone of the NPPE model.

The clinical implications of this genetic foundation are transformative. It allows us to move from a reactive model of care to a proactive, risk‐stratified one. In the near future, it may become feasible to incorporate polygenic risk scores (PRSs) in carefully selected contexts, pending further validation of predictive value, clinical utility, and equity considerations for a person newly diagnosed with epilepsy. A PRS aggregates an individual's many small genetic risk variants into a single score that quantifies their innate vulnerability to other conditions.^26^ A young man with new‐onset focal epilepsy might be found to have a high PRS for psychosis. This knowledge does not doom him to a psychotic disorder, but it acts as a crucial clinical signpost. It would prompt his care team to be hypervigilant for early psychotic symptoms, to choose an AED with a lower risk of psychiatric side effects, to educate him and his family about risk factors like cannabis use, and to schedule regular mental health check‐ins.^27^ Layer 1, the genetic blueprint, thus provides the essential starting point for truly personalized, preventive neuropsychiatric care.

### Layer 2: The epileptogenic network as a neuropsychiatric substrate—Where seizures and suffering converge

The genetic blueprint from Layer 1 does not directly cause seizures or depression. Its influence is expressed through its impact on the development and function of the brain's intricate network architecture. Layer 2 is the neurobiological heart of the NPPE model, arguing that the very same neural network that generates seizures is often the substrate for psychiatric symptoms.^3^ The distinction between the “epileptogenic zone” (the established term for the network generating seizures) and what Kanner has termed the “psychopathogenic zone” (a neologism proposed to describe the network substrate of psychiatric symptoms) is, in many cases, an artificial one.^1^

This convergence operates through several mechanisms:

#### 1. Anatomical and functional overlap

The brain is not organized into neat “epilepsy” and “psychiatry” modules. The networks are deeply interwoven. This is most evident in TLE, the most common form of focal epilepsy in adults. The structures typically involved in TLE—the hippocampus, the amygdala, the entorhinal cortex—are not merely seizure generators. They are the core components of the brain's limbic system, the central hub for emotion, memory, and motivation.^11^

The amygdala is the brain's threat detector, crucial for generating fear and anxiety. When it is chronically irritated by epileptiform activity, it is biologically inevitable that the patient will experience heightened anxiety, fear, or even panic.^28^ The hippocampus is vital for forming new memories and contextualizing experience. Its dysfunction in TLE leads not only to the classic memory deficits but also to difficulty in regulating emotional responses based on past experience, contributing to mood instability.^29^ The insula, another frequently involved structure, is the hub of interoception—the sense of the body's internal state. Its dysregulation can lead to a distorted sense of self, bodily alienation, or the somatic symptoms that are common in depression and anxiety.^30^ In TLE, therefore, anxiety and depression are not simply a psychological reaction to the seizures; they are a direct neurobiological consequence of the same network pathology that also causes the seizures.^1^

#### 2. Large‐scale network dynamics

Modern neuroscience has moved beyond a focus on individual brain regions to understanding how the brain functions through the coordinated activity of large‐scale networks.^31^ Three networks are particularly relevant to neuropsychiatry:

1. The Default Mode Network (DMN) is active during rest and self‐referential thought (mind‐wandering, thinking about the past or future). In depression, the DMN is often hyperactive and hyperconnected, reflecting a state of excessive, ruminative self‐focus.^32^
2. The Salience Network (SN), anchored in the insula and anterior cingulate cortex, detects personally relevant internal and external stimuli and switches attention between the DMN and the executive network. In anxiety and psychosis, the SN is often hyperactive, leading to a state of hypervigilance and misattribution of importance to benign stimuli.^33^
3. The Executive Control Network (ECN) is involved in goal‐directed tasks, working memory, and cognitive control.

Epilepsy profoundly disrupts the delicate balance between these networks.^34^ Epileptiform activity, even when it does not produce a clinical seizure, acts as a constant source of “noise” that interferes with normal network communication. For example, interictal spikes in the temporal lobe (a key part of the DMN) can disrupt DMN regulation, locking the patient into the same ruminative, self‐critical thought patterns seen in idiopathic depression.^35^ Abnormal firing in the insula (a key part of the SN) can trigger feelings of intense, unfounded dread or paranoia. The constant disruption of the ECN by epileptic activity contributes to the pervasive “cognitive fog” and executive dysfunction that many patients report.^36^

#### 3. The ictal–interictal continuum

The traditional sharp distinction between the “ictal” (during a seizure) and “interictal” (between seizures) state is a clinical oversimplification. Subclinical interictal epileptiform discharges (IEDs), visible on EEG but without overt clinical signs, are not benign. They represent moments of network disruption that can cause transient cognitive impairment (TCI).^37^ A student in a lecture may experience a brief IED and miss a key piece of information, or a person in a conversation may momentarily lose their train of thought. This constant, subtle disruption contributes enormously to the chronic cognitive and psychiatric burden of the disease, eroding a patient's confidence and ability to function.

#### 4. Iatrogenic network modulation

Finally, our own treatments are powerful modulators of these networks. AEDs do not just suppress seizures; they alter brain‐wide network function. Sometimes this is beneficial: Lamotrigine and valproate have mood‐stabilizing properties, but often it is detrimental.^38^ The well‐known irritability and aggression associated with levetiracetam, or the cognitive slowing and word‐finding difficulties (“Dopamax”) caused by topiramate, are direct consequences of these drugs' impact on brain networks. A clinician who prescribes an AED without considering its full network‐level psychotropic profile is flying blind.^16^

In Layer 2, we see the mind–brain divide dissolve. The epileptic network is a neuropsychiatric network. Seizures, mood, and cognition are all outputs of its dysfunction, providing a powerful, unifying framework for understanding the patient's full range of symptoms.

### Layer 3: The psychosocial vortex—A bidirectional downward spiral

The brain does not exist in a vacuum. It is in constant, dynamic interaction with the individual's psychological and social world. Layer 3 of the NPPE model posits that the relationship between the epileptic brain and the psychosocial environment is not a one‐way street but a bidirectional, self‐amplifying negative feedback loop.^1^ The brain's pathology creates profound psychosocial stress, and that psychosocial stress, in turn, feeds back to worsen the brain's pathology.

#### From brain to world: How epilepsy creates psychosocial stress

Living with epilepsy imposes a set of unique and powerful stressors that relentlessly assault a person's mental well‐being. Despite progress, epilepsy remains a highly stigmatized condition. The fear of having a seizure in public—of losing control, of being judged, of being seen as “less than”—can lead to profound social anxiety and avoidance.^39^ This leads to isolation, which is one of the strongest predictors of depression. Employment discrimination, difficulties in forming intimate relationships, and social exclusion are not just unfortunate consequences; they are powerful drivers of psychopathology.^39^, ^40^

#### Loss of control and learned helplessness

The defining feature of seizures is their unpredictability. This robs individuals of their sense of bodily autonomy and control over their own lives. This chronic state of uncertainty is the perfect incubator for anxiety.^41^ Furthermore, as articulated by psychologist Martin Seligman, when an organism is subjected to unpredictable, uncontrollable negative events, it can develop a state of learned helplessness—a passive, resigned state that is a core feature of depression. The person with epilepsy learns, through repeated experience, that their actions often have no bearing on whether a seizure occurs, leading to a deep‐seated sense of futility.^42^

#### The burden of treatment and cognitive deficits

The daily grind of medication adherence, frequent medical appointments, and managing side effects is itself a chronic stressor. Furthermore, the cognitive deficits associated with both the epilepsy and the AEDs—particularly in memory, attention, and executive function—create constant friction in daily life, leading to failure and frustration at school, at work, and in relationships.^9^

#### From world to brain: How stress worsens epilepsy

The crucial insight of the NPPE model is that this causal arrow also points in the opposite direction. Psychosocial stress is not just a consequence of epilepsy; it is a potent neurobiological seizure trigger.^43^

#### The neurobiology of stress

When a person experiences psychological stress, their brain activates the hypothalamic–pituitary–adrenal (HPA) axis, culminating in the release of the stress hormone cortisol. While adaptive in the short term, chronic stress leads to chronically elevated cortisol levels.^44^ Cortisol has direct effects on the brain: it enhances the activity of excitatory neurotransmitters like glutamate and dampens the activity of inhibitory neurotransmitters like gamma‐aminobutylic acid (GABA). This combination pushes the brain toward a state of neuronal hyperexcitability, effectively lowering the seizure threshold. Thus, the stress caused by worrying about seizures can, through a concrete biological pathway, make seizures more likely.^45^

#### Behavioral pathways

Stress also impacts behavior in ways that increase seizure risk. A stressed individual is more likely to experience poor sleep, and sleep deprivation is one of the most well‐known seizure triggers. They may be more likely to forget to take their medication, use alcohol or other substances to cope, or eat poorly. Each of these behaviors can destabilize the brain and provoke a seizure.^46^

This bidirectional loop creates a vicious cycle. The epilepsy causes stress, which in turn worsens the epilepsy, which causes more stress. The patient becomes trapped in a downward spiral where their brain and their life circumstances conspire to undermine their well‐being. Breaking this cycle requires interventions that target both the brain (Layer 2) and the psychosocial environment (Layer 3) simultaneously.^16^

### Layer 4: The fractured system—Societal and institutional amplifiers

An individual does not exist in isolation. Their personal feedback loops of brain and stress are embedded within a much larger system of social, institutional, and political structures. Layer 4 of the NPPE model argues that these macro‐level systems often act as powerful amplifiers of patient suffering, creating structural barriers to recovery and reinforcing the problems found in the other layers.

#### The fractured healthcare system

The most immediate system impacting the patient is the healthcare system itself, which, as discussed earlier, is foundationally fractured. The institutional separation of neurology and psychiatry is a primary driver of fragmented care.^6^ Separate clinics, separate medical records, separate training programs, and separate professional cultures create a system where comprehensive, integrated care is almost impossible to deliver. This is further exacerbated by healthcare reimbursement policies. A fee‐for‐service model incentivizes short, high‐volume appointments focused on a single problem (e.g., medication adjustment), while discouraging the longer, more complex consultations required for true neuropsychiatric integration.^47^

#### Legal and regulatory frameworks

Society's response to epilepsy is codified in laws and regulations that, while often intended to protect public safety, can become major sources of chronic stress and disability. Driving regulations are a prime example. The loss of a driver's license is not an inconvenience; in many societies, it represents a profound loss of independence, employability, and social connection.^48^ The fear of losing one's license can lead patients to conceal seizures from their doctors, undermining their own care. Similarly, while laws like the Americans with Disabilities Act (ADA) exist to prevent employment discrimination, the practical reality is that many employers are hesitant to hire someone with a known seizure disorder, forcing individuals into unemployment or underemployment, with devastating financial and psychological consequences.^40^

#### The educational system and public awareness

The cycle of stigma and misunderstanding is perpetuated by a failure of public education. When teachers, employers, and the general public are poorly informed about epilepsy, they react with fear and prejudice.^39^ This lack of knowledge in the educational system means that a child with epilepsy may not receive the specific academic accommodations they need for their cognitive difficulties. A lack of public awareness, often fueled by sensationalized media portrayals, reinforces the social isolation (Layer 3) and systemic barriers (Layer 4) that plague the lives of people with epilepsy.^39^

Layer 4 reveals that individual suffering cannot be fully understood or treated without addressing the societal context in which it occurs. A truly holistic approach must involve not only treating the individual patient but also advocating for systemic change—integrated‐care models, fairer laws, and better public education—to create an environment that supports, rather than amplifies, the struggle of living with epilepsy.

### The interplay of the four layers

The NPPE framework is not merely a static, hierarchical list; it is a dynamic system characterized by continuous, bidirectional interactions between the layers. Understanding these feedback loops is essential to appreciating the model's explanatory power.

- From genes to brain (Layer 1 → Layer 2). The causal cascade begins with the genetic blueprint. A shared genetic vulnerability (Layer 1) influences the development and function of neural circuits, increasing the probability of network instability. This shapes the very architecture of the epileptogenic network (Layer 2), predisposing it to generate not only seizures but also psychiatric symptoms.
- From brain to experience (Layer 2 → Layer 3). The dysfunctional network (Layer 2) is the engine of symptomatology. It produces seizures, cognitive deficits, and direct neurobiological drivers of altered mood and anxiety. These phenomena directly create the profound psychosocial stressors of Layer 3, such as the fear of future seizures, the frustration of cognitive fog, and the experience of depression or anxiety.
- From experience back to brain (Layer 3 → Layer 2). This is a critical feedback loop. The psychosocial stress generated in Layer 3 is not just an experiential outcome; it is a potent biological agent. Through mechanisms like the HPA axis and the release of cortisol, chronic stress directly modulates neuronal excitability, lowering the seizure threshold and further destabilizing the epileptogenic network (Layer 2). This creates a vicious cycle where anxiety about seizures can biologically increase the likelihood of having them.
- From system to person (Layer 4 → Layer 3). The societal and institutional structures of Layer 4 act as powerful amplifiers of individual distress. Systemic barriers like driving restrictions, employment discrimination, and a fragmented healthcare system directly create and intensify the psychosocial stressors of Layer 3. A person's loss of a driver's license (Layer 4) is not an abstract policy issue; it is a direct cause of social isolation and vocational disability (Layer 3).
- From person back to system (Layer 3 → Layer 4). This loop also operates in reverse. For instance, a patient experiencing high levels of seizure‐related anxiety (Layer 3) may conceal their seizures from clinicians to avoid driving restrictions (Layer 4), which undermines their own care and perpetuates the cycle of poor seizure control.

By detailing these interactive pathways, NPPE provides a more comprehensive and dynamic picture of how psychiatric comorbidities in epilepsy are generated and sustained, offering multiple potential points for targeted, multi‐level intervention.

## CHARTING A NEW COURSE—THE CLINICAL AND RESEARCH IMPLICATIONS OF NPPE

The clinical components described herein (e.g., PRS, rs‐functional magnetic resonance imaging [MRI], EEG, and magnetoencephalography [MEG]) are exploratory and, in most settings, not yet feasible for routine care due to cost, access, and insufficient validation. We frame them as candidate tools to be evaluated in research or specialized tertiary contexts, not as current standards of care. The NPPE paradigm is not merely a descriptive model; it is a prescriptive one. It provides a blueprint for a radical transformation in both the clinical practice and the research agenda for the psychiatric dimensions of epilepsy.^8^, ^27^ It moves us from a paradigm of labeling symptoms to one of understanding mechanisms, and from a one‐size‐fits‐all approach to one of multi‐level, personalized intervention.^19^

### A proposed model for clinical practice—Toward fragmentation to integration

It must be stated unequivocally that the NPPE paradigm, as outlined, is a theoretical proposal and a call for research, not a validated clinical protocol. The following sections describe a hypothetical model of how clinical practice could be structured if this framework were adopted. The efficacy, feasibility, and cost‐effectiveness of the NPPE model are hypotheses that require rigorous testing through prospective longitudinal cohort studies and, eventually, randomized controlled trials before any widespread clinical implementation. Adopting the NPPE framework could potentially reshape the clinical encounter, aiming to create a more comprehensive, mechanistic, and effective model of care.^3^

#### The NPPE integrated assessment

**Figure 1:**
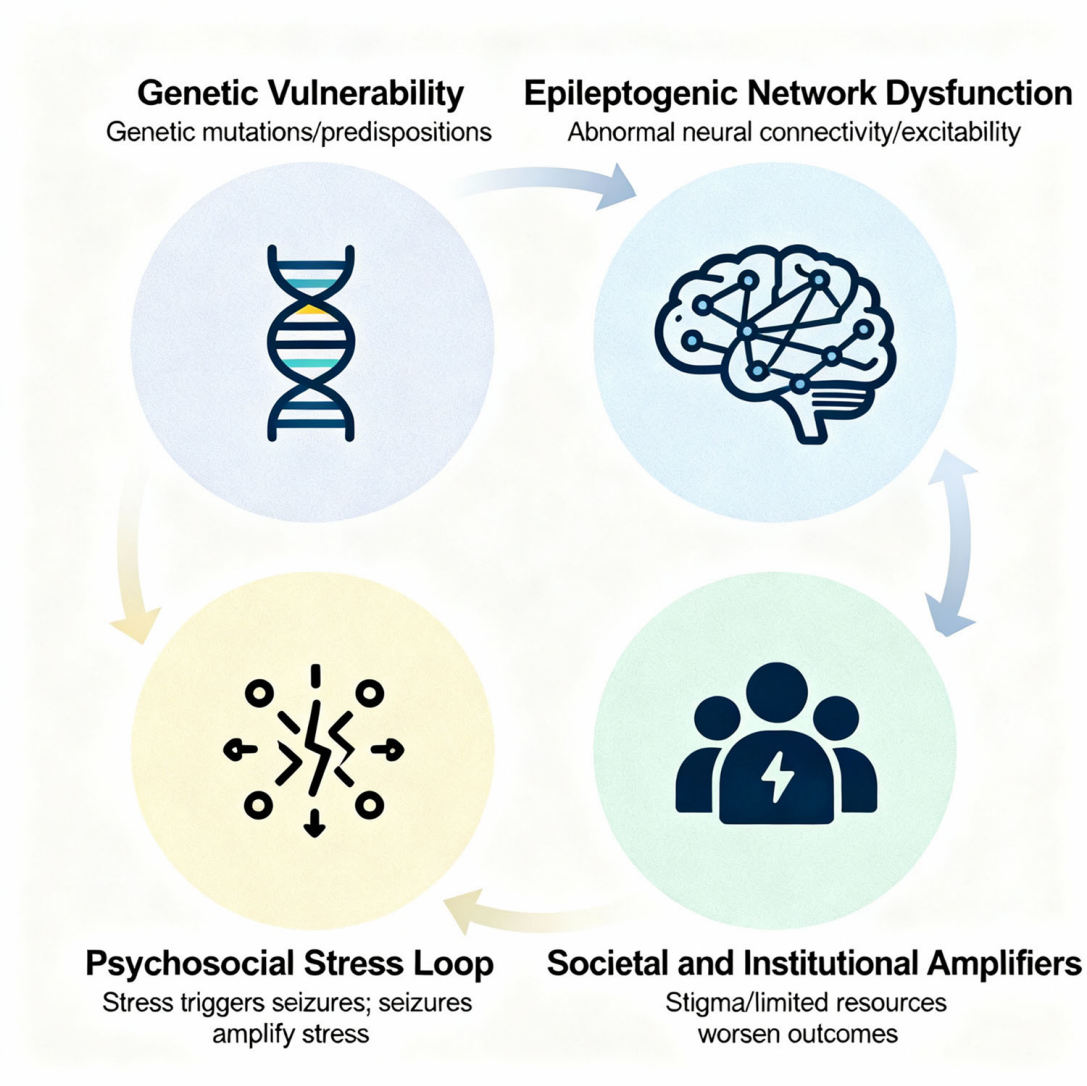
The four‐layer Network‐Pluralistic Psychiatry in Epilepsy (NPPE) model. This diagram illustrates the four hierarchical and interacting causal layers of the NPPE framework. The model integrates (1) foundational genetic vulnerability, (2) core epileptogenic network dysfunction, (3) the resulting psychosocial stress loop experienced by the patient, and (4) the broader societal and institutional amplifiers that shape the illness. The arrows indicate the dynamic, bidirectional interactions between layers, highlighting how factors at each level influence and are influenced by the others to produce the overall clinical presentation.

A primary implication of the NPPE model would be a new approach to assessment. This framework suggests the value of a single, integrated neuropsychiatric assessment that gathers data across all four layers. A hypothetical “NPPE Initial Assessment Protocol” might look like Figure 1.

#### Mechanistic, integrated therapeutics

Armed with this integrated assessment, the clinical team can design interventions that target specific mechanisms across multiple layers. Each proposed intervention is a candidate mechanism‐targeted approach whose safety, efficacy, and cost‐effectiveness must be established in controlled studies prior to broader adoption. Off‐label or device‐based strategies should be confined to protocols with appropriate oversight.

#### Rational pharmacotherapy

Under this model, medication choices could become a more data‐driven exercise in “network pharmacology.”^10^ For a patient with TLE, anxiety, and a genetic variant affecting GABAergic function, a medication that enhances GABAergic tone (like a benzodiazepine or valproate) might be considered, weighing its antiepileptic and anxiolytic benefits against its side effects. For a patient with depression, lamotrigine might be a superior first‐choice AED due to its known mood‐stabilizing properties.^16^ Pharmacogenomic data would guide dosing and predict adverse reactions, moving beyond the current trial‐and‐error approach.^27^

#### Epilepsy‐specific psychotherapies

Therapy would be tailored to the unique challenges of the condition. This would involve the development and dissemination of manualized therapies like “CBT for Epilepsy (CBT‐E)” or “Acceptance and Commitment Therapy for Epilepsy (ACT‐E).”^49^ These therapies would include core modules on the following:

- Psychoeducation about the NPPE model itself, helping patients understand the brain–mind–life connection and destigmatizing their psychiatric symptoms.
- Techniques for managing the fear of seizures and reducing safety‐seeking behaviors that constrict life.
- Strategies for coping with cognitive side effects of AEDs.
- Interventions to challenge internalized stigma and rebuild a positive self‐identity.
- Behavioral strategies for managing seizure triggers like stress and poor sleep.^50^

#### Network‐targeted interventions

The NPPE model opens the door to using neuromodulation techniques with a dual purpose. Devices like the Vagus Nerve Stimulator (VNS), already approved for both epilepsy and treatment‐resistant depression, would be seen as a primary network‐modulating therapy.^51^ Transcranial magnetic stimulation (TMS) could be targeted at specific nodes of dysfunctional networks (e.g., inhibiting a hyperactive DMN node in the prefrontal cortex) to simultaneously improve mood and potentially disrupt the spread of epileptiform activity.^52^

#### Integrated‐care models

The ultimate clinical expression of the NPPE paradigm is the breakdown of institutional silos. This means creating integrated epilepsy care centers where neurologists, psychiatrists, psychologists, neuropsychologists, specialist nurses, and social workers function as a single, co‐located team.^47^ This team would conduct integrated assessments, hold joint case conferences, and develop a single, unified treatment plan, ensuring that the patient's brain, mind, and life are treated as the inseparable whole they truly are.^21^

#### Conceptual novelty: Positioning NPPE relative to previous pluralistic approaches

The NPPE framework is explicitly pluralistic, building on the intellectual foundations of earlier models like the BPS model and the NIH's Research Domain Criteria (RDoC).^53^ However, NPPE offers a critical innovation by providing a mechanistically specified and disease‐contextualized framework specifically for epilepsy, thereby moving beyond the limitations of these vital but more general approaches.

- Advancing beyond the BPS model. The BPS model provides an indispensable philosophical orientation toward whole‐person care but is often criticized for being a high‐level heuristic that does not, by itself, specify the causal pathways or dynamic interactions between its domains. NPPE operationalizes the BPS philosophy into a testable, multi‐level model for epilepsy. It moves beyond the generic categories of “bio,” “psycho,” and “social” by positing a concrete, directional, and testable causal cascade: how specific genetic liabilities (Layer 1) modulate epileptogenic networks (Layer 2), which in turn create and are reciprocally influenced by psychosocial dynamics (Layer 3) and systemic barriers (Layer 4). It transforms a philosophical stance into a scientific research program.
- Contextualizing the RDoC framework. NPPE embraces the trans‐diagnostic, multi‐level spirit of RDoC. However, RDoC is intentionally agnostic to traditional disease categories in its mission to uncover fundamental dimensions of psychopathology. In contrast, NPPE is firmly anchored in the known neuropathology of a specific disease: epilepsy. It proposes that the epileptogenic network itself (Layer 2) acts as a primary organizing hub that shapes and constrains psychiatric expression. This disease‐specific focus allows NPPE to generate unique, testable hypotheses that a disease‐agnostic framework cannot, for example, predicting distinct psychiatric phenotypes based on the anatomical location and connectivity of a patient's seizure network. In this way, NPPE serves as a crucial bridge, demonstrating how the mechanistic depth of RDoC can be fruitfully applied within a complex and specific neuropsychiatric disease context.

### A new research agenda—From description to mechanism

To realize the full potential of the NPPE paradigm, a new direction in research is required. The field must move beyond descriptive studies that simply document the high rates of comorbidity and toward mechanistic studies that test the causal pathways proposed by the NPPE model.^19^

#### Launch longitudinal, multimodal cohort studies

The field needs large‐scale, long‐term studies that follow individuals from their first seizure, collecting data across all four NPPE layers over time. This would allow researchers to track how genetic predispositions interact with developing brain networks and environmental stressors to produce specific neuropsychiatric outcomes.^3^

#### Integrate network biomarkers into clinical trials

Future clinical trials for new AEDs or psychosocial interventions should include network‐level biomarkers (e.g., changes in functional magnetic resonance imaging [fMRI] or EEG connectivity) as key outcome measures.^31^, ^54^ This would allow us to determine how an intervention works, not just if it works. A trial for a new drug should not just ask, “Does it reduce seizure frequency?” but also, “Does it normalize DMN hyperactivity?” and “Does it improve cognitive network efficiency?”^36^

#### Develop and test integrated‐care models

Health services research is urgently needed to design, implement, and evaluate the effectiveness and cost‐effectiveness of the integrated‐care models proposed above.^47^ This research must demonstrate to policymakers and healthcare funders that while integrated care may be more resource‐intensive upfront, it leads to better long‐term outcomes and lower overall system costs by preventing crises and improving functional recovery.^40^

#### Focus on trans‐diagnostic mechanisms

Research should shift from a focus on traditional diagnostic categories to studying trans‐diagnostic mechanisms, similar to the RDoC framework.^10^ For example, instead of studying “depression in epilepsy,” researchers could study “anhedonia in epilepsy,” investigating its shared and distinct mechanisms (e.g., reward circuit dysfunction) across different epilepsy syndromes and comparing them to idiopathic anhedonia.^3^ This would build a more robust, biologically grounded understanding of psychiatric symptoms.

### Limitations and considerations for clinical implementation

While the NPPE framework provides a comprehensive conceptual map, its translation into a routine clinical protocol faces significant practical challenges that must be acknowledged. The full assessment battery outlined in Table 1 represents an idealized state and is not intended as a universal prescription for every patient.

**Table 1:** Network‐Pluralistic Psychiatry in Epilepsy (NPPE) integrated assessment (hypothesis‐generating, tiered; research‐use or tertiary‐care contexts only).

NPPE layer	Assessment tools and methods	Information gained
Layer 1: genetic	– Comprehensive family history of neurological and psychiatric illness – Pharmacogenomic panel (e.g., for CYP450 enzymes)^27^ – (Future) polygenic risk score calculation for depression/psychosis^26^ – Targeted genetic testing if indicated^21^	– Innate vulnerability profile – Guidance for initial AED selection – Prediction of medication metabolism and side effect risk
Layer 2: network	– High–density EEG and/or MEG to map epileptiform network – Structural MRI (e.g., for subtle dysplasia or hippocampal sclerosis) – (Research/future) resting‐state fMRI to assess DMN/SN/ECN connectivity^36^ ‐ Comprehensive neuropsychological testing^29^	– Localization of seizure network – Objective evidence of network dysfunction – Baseline cognitive strengths/weaknesses – Biomarker for treatment response
Layer 3: psychosocial	– Epilepsy–specific QOL scales (e.g., QOLIE–31)^12^ – Standardized scales for depression (PHQ‐9), anxiety (GAD‐7) – Stigma scales (e.g., Epilepsy Stigma Scale)^39^ ‐ Assessment of coping strategies, social support, learned helplessness^42^ ‐ (Future) biomarkers of stress (e.g., salivary cortisol)^45^	– Patient's subjective experience of illness burden – Identification of key psychological targets for therapy – Understanding of the patient's resilience factors and stressors
Layer 4: systemic	– Assessment of social determinants of health (housing, employment, finances)^47^ – Evaluation of access to care, transportation barriers – Understanding of legal challenges (driving, employment)^48^ – Mapping of patient's social/community support network	– Identification of structural barriers to care and recovery – Targets for social work, case management, and advocacy – Understanding the patient's broader life context

*Note*: Polygenic risk score (PRS), rs‐fMRI, EEG, and MEG are investigational for psychiatric stratification in epilepsy; predictive validity, cost‐effectiveness, and clinical utility remain to be established. Not intended for routine clinical use.

Abbreviations: AED, antiepileptic drug; DMN, Default Mode Network; ECN, Executive Control Network; EEG, electroencephalogram; fMRI, functional magnetic resonance imaging; GAD‐7, Generalized Anxiety Disorder‐7; MEG, magnetoencephalography; MRI, magnetic resonance imaging; PHQ‐9, Patient Health Questionnaire‐9; QOL, quality of life; SN, Salience Network.

#### Feasibility, cost, and resource allocation

The most immediate barriers are feasibility and cost. Advanced modalities such as high‐density EEG/MEG, resting‐state fMRI, and comprehensive genetic panels are resource‐intensive, expensive, and not widely available outside of specialized academic medical centers. Proposing their routine use for all patients with epilepsy is currently unrealistic and would strain most healthcare systems. This raises the critical issue of patient selection and the potential for iatrogenic effects, where the burden of excessive testing could increase patient anxiety and financial distress without a clear clinical benefit.

#### A proposed stepped‐care model and illustrative case

To address these practical barriers, a pragmatic approach would involve a stepped care or tiered implementation of the NPPE assessment, where the intensity of investigation is scaled to clinical complexity. To illustrate this, let us consider a hypothetical case.

The Case of Mr. A: Mr. A is a 28‐year‐old company worker recently diagnosed with left TLE.

#### Tier 1 (universal assessment)

Mr. A's initial visit triggers the universal Tier 1 assessment. A comprehensive history reveals a family history of anxiety (his father had “bad nerves”), providing an early clue for Layer 1 vulnerability. Standard screening tools (Layer 3) show a PHQ‐9 score of 14 (moderate depression) and a Generalized Anxiety Disorder‐7 score of 12 (moderate anxiety). He reports catastrophic fears about having a seizure at work and expresses hopelessness about his career. An assessment of Layer 4 barriers reveals significant anxiety related to driving restrictions and potential workplace stigma.

##### Outcome

This low‐cost, foundational assessment successfully identifies a significant neuropsychiatric comorbidity and its psychosocial context, immediately triggering an escalation to Tier 2.

#### Tier 2 (assessment for comorbidity)

Because Mr. A screened positive and his symptoms are impacting his QOL, his care is escalated.

A comprehensive neuropsychological evaluation (Layer 2) is conducted. It confirms the expected verbal memory deficits associated with his left TLE but also reveals impaired attention and processing speed, which are likely exacerbated by his depression.

A pharmacogenomic panel (Layer 1) is ordered to guide pharmacotherapy. The results indicate he is an intermediate metabolizer for the CYP2C19 enzyme, suggesting that standard doses of some SSRIs (e.g., sertraline) may be less effective.

##### Integrated treatment plan

The Tier 2 data allow for a mechanism‐informed, personalized plan. The care team initiates an AED with a favorable psychotropic profile and refers to Mr. A for an epilepsy‐specific psychotherapy (e.g., CBT‐E) to directly target his illness‐related anxiety (Layer 3). The choice of antidepressant is guided by the pharmacogenomic findings to optimize efficacy.

#### Tier 3 (assessment for complex, refractory cases)

Initially, Mr. A's case does not require Tier 3 intervention. However, let us imagine his seizures become medically refractory and his depression deepens, accompanied by transient paranoid ideation. He is now a candidate for a pre‐surgical evaluation, necessitating escalation to Tier 3.

Research‐grade neuroimaging (Layer 2), including resting‐state fMRI, is performed. The fMRI reveals hyperactivity in the DMN (correlating with his depressive rumination) and aberrant connectivity in the SN (a potential substrate for his paranoia). High‐density EEG combined with MEG is used to map his epileptogenic network, revealing that the seizure onset zone anatomically overlaps with the dysfunctional nodes of his DMN.

A research‐based PRS for psychosis (Layer 1) is calculated and found to be high, providing a biological context for his paranoid thinking and informing the team to use antipsychotic medications with extreme caution.

##### Synthesized decision

The Tier 3 data provide a unified neuropsychiatric picture. The anatomical overlap between the seizure network and the mood‐regulating network explains why his mood and seizure control are so intertwined. Based on this multilayered understanding, the clinical team prioritizes a less invasive, mechanism‐targeted intervention—rTMS aimed at modulating the hyperactive DMN node—as a first step before reconsidering resective surgery. This case demonstrates how the stepped‐care model allows for a scalable, resource‐appropriate, and mechanistically integrated approach to care.

#### The challenge of data synthesis: From pluralism to a unified clinical decision

A final, and perhaps most profound, challenge lies in synthesizing the multi‐level information gathered. The pluralism of the NPPE framework is both its greatest strength and its most significant practical weakness. It avoids simplistic reductionism but, in doing so, risks creating a new form of “atheoretical eclecticism,” where a clinical team is overwhelmed by a flood of data without a clear method for integrating it into a coherent treatment plan. Arguing what data to collect is insufficient; we must also propose how to synthesize it.

To move beyond this impasse, the NPPE model necessitates new clinical processes. A hypothetical decision‐making workflow might involve a multidisciplinary NPPE case conference. Consider the example of a patient in Tier 2 with a high polygenic risk for psychosis (Layer 1), subtle hyperactivity in the SN on fMRI (Layer 2), and reports of significant social stigma driving withdrawal (Layer 3). How should a team prioritize these factors?

The NPPE conference would bring together the neurologist, psychiatrist, psychologist, and social worker to collaboratively weigh these findings. The team might conclude that while the genetic risk (Layer 1) is a non‐modifiable background factor and the network hyperactivity (Layer 2) is a potential target for future neuromodulation, the most immediate and modifiable driver of distress is the social stigma and withdrawal (Layer 3). Therefore, the initial intervention would prioritize an evidence‐based psychotherapy (e.g., ACT or CBT) focused on challenging internalized stigma and promoting social re‐engagement. Simultaneously, the pharmacotherapy plan would be informed by the Layers 1 and 2 data, leading the team to consciously avoid antipsychotics that might lower the seizure threshold and instead choose an AED with a more favorable psychotropic profile. This process transforms a list of disparate data points into a prioritized, sequenced, and mechanism‐informed treatment strategy.

In the long term, these human‐led clinical reasoning processes could be augmented by the development of computational decision‐support tools. The ultimate goal of future research, therefore, is not just to validate the individual layers of the NPPE model, but to create and rigorously test the evidence‐based heuristics and clinical algorithms needed for their integration. This research must focus on determining the relative weighting of different factors across various clinical presentations, eventually providing clinicians with a probabilistic framework to guide personalized treatment.

## CONCLUSION: RECLAIMING THE BORDERLAND

The psychiatric suffering of people with epilepsy is not a footnote to their neurological condition; it is a central chapter in their story.^1^, ^10^ For too long, our systems of care, blinded by the twin dogmas of neurological reductionism and psychiatric formalism, have forced patients to tear this story in two, presenting their brain to one specialist and their mind to another.^6^ It has left millions in a state of fragmented, ineffective care, their suffering amplified by a system that refuses to see them as whole.^3^

The NPPE paradigm offers a way forward.^8^, ^19^ It is a call to dissolve the artificial boundaries between disciplines and to build a new science and practice of neuropsychiatry grounded in the interconnected realities of genes, brain networks, psychosocial stress, and societal structures.^3^, ^9^ It provides a framework to move beyond simply labeling symptoms and toward understanding the complex, multi‐level mechanisms that produce them. NPPE outlines a path toward a more principled, data‐driven approach; however, achieving this goal depends on future studies that validate, refine, or refute its core tenets.^8^, ^27^

Implementing this paradigm will not be easy. It requires a fundamental rethinking of our clinical workflows, our research priorities, and our educational curricula.^47^ It demands a new generation of clinicians and scientists who are fluent in the languages of both neuroscience and human experience.^3^ Most of all, it requires intellectual humility to admit that our old maps of the mind and brain are no longer adequate, and the scientific rigor to empirically test the new ones we propose. The critical next step is to move this framework from theory to hypothesis, and to begin the systematic research needed to validate, refine, or refute its core tenets.

The borderland between neurology and psychiatry is not an empty space; it is the place where our patients live their entire lives.^4^ It is our ethical and scientific imperative to reclaim this territory, to explore it with the best tools we have, and to build a system of care that is as integrated, complex, and whole as the human beings it is meant to serve.^3^

## AUTHOR CONTRIBUTIONS

N/A.

## CONFLICT OF INTEREST STATEMENT

The author declares no conflicts of interest.

## ETHICS APPROVAL STATEMENT

N/A.

## PATIENT CONSENT STATEMENT

N/A.

## CLINICAL TRIAL REGISTRATION

N/A.

## Data Availability

Data sharing is not applicable to this article as no datasets were generated or analyzed during the current study.
